# Using an Innovative Bifunctional Siloxane to Protect Cement Composite Surfaces from Biological Corrosion

**DOI:** 10.3390/ijms26115052

**Published:** 2025-05-23

**Authors:** Marta Thomas, Joanna Karasiewicz, Paulina Nowicka-Krawczyk, Rafał M. Olszyński, Łucja Balcerzak, Miłosz Frydrych, Bogna Sztorch, Agnieszka Ślosarczyk

**Affiliations:** 1 Faculty of Civil and Transport Engineering, Institute of Building Engineering, Poznan University of Technology, 60-965 Poznan, Poland; marta.thomas@put.poznan.pl (M.T.); agnieszka.slosarczyk@put.poznan.pl (A.Ś.); 2 Faculty of Chemistry, Adam Mickiewicz University in Poznań, Uniwersytetu Poznańskiego 8, 61-614 Poznan, Poland; 3 Department of Algology and Mycology, Faculty of Biology and Environmental Protection, University of Lodz, Banacha 12/16, 90-237 Lodz, Poland; paulina.nowicka@biol.uni.lodz.pl (P.N.-K.);; 4 Laboratory of Microscopic Imaging and Specialized Biological Techniques, University of Lodz, Banacha 12/16, 90-237 Lodz, Poland; 5 Centre for Advanced Technologies, Adam Mickiewicz University in Poznań, 61-614 Poznan, Poland

**Keywords:** functionalized siloxane, surface modification, algae, cement composite, biocorrosion

## Abstract

This study tested the effectiveness of a bifunctional polysiloxane (L43) as a means of protecting concrete surfaces from biocorrosion. L43 was designed to contain two types of functional groups in its structure: surface-active hydrophobic chains and hydrophilic groups that allow the coating to permanently bond to the concrete. L43-coated cement samples achieved compressive strengths exceeding 70 MPa, while samples subjected to cyclic freeze–thaw tests achieved compressive strengths exceeding 33 MPa. In addition, compound L43 at a concentration of 5% reduced the photosynthetic activity of microalgae cells on the concrete surface. The maximum value of chlFI decreased by 69.5%, while the average value decreased by 71.4%. Thus, it was proven that compound L43 effectively counteracts biological corrosion without deteriorating the structure of the impregnated substrate. It should be emphasized that the biocidal effect is due to the structure of the siloxane compound and appropriately selected functional groups. There is no need to add harmful biocides, making the solution environmentally friendly. In addition, the coating allows for free air circulation, which is crucial for the protection of building and construction materials.

## 1. Introduction

The growth of algae on the surface of building materials is an undesirable phenomenon with negative aesthetic, structural issues, and health-related impacts. The most noticeable effect is the appearance of unsightly discoloration and stains on the material’s surface, necessitating frequent cleaning and maintenance [1,2,3]. The appearance of stains reduces the aesthetic value of buildings, which negatively affects their perception, especially in the case of historic structures [4]. Another negative effect is the toxins and allergens released by algae, which can be harmful to human health [5,6]. Moreover, algae, as the pioneer colonizers, enhance the microbial succession of heterotrophic components—bacteria and fungi—and together they form a complex biofilm matrix with high corrosive potential. Microbial-influenced corrosion (biological corrosion) is a complex phenomenon involving the interaction between bacteria, fungi, and phototrophic microorganisms in multi-species biofilms and the surfaces they colonize, leading to gradual degradation of materials [7]. Such algal component biofilms locally alter pH and oxygen levels, which promotes the activity of sulfate-reducing bacteria and the production of sulfuric acid. This process aggressively attacks both the cement matrix and the steel reinforcement [6]. Algae secrete low-molecular-weight organic acids, which chemically degrade hydrated cement components like portlandite and ettringite. This results in the dealkalization of the matrix and a loss of cohesive strength [8,9]. Additionally, the diurnal cycles of photosynthesis and respiration by algae create differential aeration cells at the interface between concrete and steel, significantly enhancing the electrochemical corrosion rates of the reinforcement. Through their metabolic uptake of cement constituents, such as calcium, magnesium, and silica, they induce the formation of microfissures and cracks, which compromise the integrity of the mortar and increase its permeability [6,8]. Detachment of biofilm crusts and patinas increases surface porosity and moisture retention, revealing the inner layers of material for successive colonization, which perpetuates a cycle of biodeterioration [9]. The negative impact on the material’s structure is therefore highly significant, as the development of microorganisms leads to biodeterioration, resulting in both physical and chemical changes in the material’s structure [3,10,11,12,13,14,15].

As the concrete structures are susceptible to microbially induced corrosion, which threatens their durability and safety, it is essential to find a solution to stop the rate of microbial colonization. To combat this issue, biochemical strategies target microbial metabolism and the local chemistry to inhibit corrosion. For instance, D-tyrosine, tetrakis (hydroxymethyl)phosphonium sulfate (THPS), and titanium dioxide (TiO_2_) nanoparticles can each suppress sulfur-oxidizing bacteria [16]. Concrete modified with nano-TiO_2_, nano-calcium carbonate (CaCO_3_), and a sodium nitrite (NaNO_2_)-based inhibitor demonstrates minimal pH drop and limited bacterial attachment in accelerated corrosion tests [17]. Furthermore, treating the concrete with a combined solution of glutaraldehyde and didecyl-dimethyl-ammonium chloride not only eradicates fungi but also enhances the mechanical integrity of the cement matrix [18]. Incorporating zinc oxide (ZnO) into cementitious coatings significantly reduces algal adhesion and subsequent sulfuric acid attacks, outperforming traditional biocides [19]. However, the use of biocides has a negative environmental impact due to their leaching into the soil [20]. Additionally, biocides pose direct and indirect health risks to humans by increasing the release of mycotoxins from moulds, and contact with biocides may cause allergic skin reactions [21,22]. Moreover, leaching significantly reduces their effectiveness [22]. The negative environmental impact of biocides has led to the introduction of legal restrictions on their use. Furthermore, although biocides should be registered, there are still insufficient testing procedures for materials used in construction [1,22].

Therefore, it is necessary to explore new agents that can limit the biocorrosion of building materials. Silicone-based compounds are widely used in construction due to their non-toxicity and biodegradability, making them a safe and environmentally friendly alternative [23,24]. Moreover, silicone-based agents exhibit thermal and oxidative stability as well as low reactivity, making them durable and resistant to even extreme weather and chemical conditions [23]. Additionally, silanes can effectively protect cement-based building materials from water, chloride ions, deicing salts, and freeze–thaw cycles, thereby safeguarding and improving the material’s durability [24,25,26]. Equally important is the ability of silane to form a hydrophobic layer, which, by penetrating deep into the cement composite, effectively blocks the ingress of harmful substances [24,26]. The development of such widely applicable agents is made possible by modifying the molecular chain structure of silane.

The authors decided to modify the structure of siloxane in such a way as to obtain a durable surface protection agent for cement composites against biocorrosion. This publication presents results on the effectiveness of this agent in preventing biocorrosion growth on the protected surface of the cement composite for two methods of applying the impregnant, as well as the impact of the compound application on the physicomechanical properties of the cement composites.

## 2. Results

### 2.1. Synthesis Results

**Figure 1:**
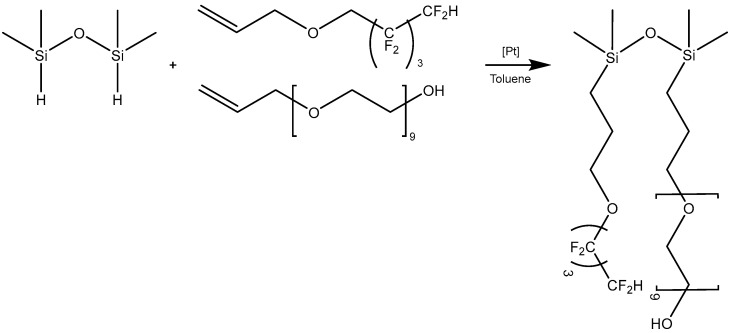
Synthesis of bifunctional compound L43 by hydrosilylation reaction.

The method of successive hydrosilylation with two different olefins was used to introduce functional groups into the system in a controlled manner. This method makes it possible to obtain a derivative with precisely defined surface parameters. The hydrophobic nature of the siloxane chain was enhanced by the presence of a fluoroalkyl substituent, which additionally reduced the surface tension of the obtained derivative. The hydrophilic nature of the obtained compound was obtained by introducing polyether compounds, which easily form hydrogen bonds with water molecules in solution due to the presence of oxygen in the ethylene bridges. By introducing two substituents with extreme surface properties—hydrophilic (polyether groups) and hydrophobic (fluoroalkyl groups)—a compound with amphiphilic properties was obtained, as shown in Figure 1. The backbone structure makes silicone compounds permeable to water vapor and gases, which is crucial for the protection of building materials.

Product characterization:
**^1^H NMR (CDCl_3_) δ (ppm):** −0.08 (6H, (Si(CH_2_)CH_3_); −0.04 (18H Si(CH_3_)_3_); 0.40 (4H, SiCH_2_); 1.49 (4H, SiCH_2_CH_2_); 3.31 (4H, SiCH_2_CH_2_CH_2_); 3.51 (18H, OCH_2_CH_2_), 3.66 (18H OCH_2_CH_2_), 6.01 (1H, OH); 7.26 (1H, CF_2_H)
**^13^C NMR (CDCl_3_) δ (ppm):** −0.05 (Si(CH_2_)CH_3_); 0.88 (Si(CH_3_)_2_); 13.94 (SiCH_2_CH_2_CH_2_), 23.06 (SiCH_2_CH_2_CH_2_); 61.39 (OCH_2_CH_2_) 64.15 (CH_2_O(CF_2_)_3_CF_2_H); 70.33 (OCH_2_CH_2_); 105.78 (OCF_2_CF_2_CF_2_CF_2_H); 107.46 (OCF_2_CF_2_CF_2_CF_2_H); 109.14 (OCF_2_CF_2_CF_2_CF_2_H); 115.29 (OCF_2_CF_2_CF_2_CF_2_H);
**^29^Si NMR (CDCl_3_) δ (ppm):** 7.67 (Si(CH_3_)_3_); −20.87 (Si(CH_3_)_2_);

The thermogravimetry of the product was also investigated. In the temperature range up to 200 °C, the first weight loss is observed due to the evaporation of solvents and other volatile fractions, which indicates the probable beginning of sample decomposition or evaporation (by 24.35%). The next weight loss is indicative of the thermal decomposition of the sample (by 73.97%) up to approximately 500 °C. In the temperature range of 500−800 °C, the weight loss is minimal (1.085%) (Supplementary Material Figure S1).

Differential scanning calorimetry (DSC) analysis of the synthesized compound was also performed. In the first cycle of heating and cooling of the sample, an endothermic melting signal (−3.66 °C) and an exothermic solidification signal (−19.97 °C) are visible. There are no additional signals indicating other thermal processes taking place in the sample. In the second cycle of the thermogram, only the endothermic signals from melting (4.14 °C) and the exothermic signal from crystallization (−19.97 °C) were observed (Supplementary Material Figure S2).

### 2.2. Physical and Mechanical Properties of Coated Cement Samples

#### 2.2.1. Density

The volumetric density of the samples coated with L43 by dipping-D and painted-P is comparable to the volumetric density of the samples without applied compounds. The density of the reference sample was 2.166 ± 0.008 g/cm^3^, while that of the dip-coated sample was 2.118 ± 0.004 g/cm^3^, and that of the sample covered by painting was 2.129 ± 0.006 g/cm^3^.

#### 2.2.2. Water Absorption

For specimens to which the formulation was surface-applied by dipping or painting, the water absorption is comparable to that of specimens without the formulation applied. It can be assumed that the water absorption of the cementitious matrix is not affected by applying L43 on the surface of the cement samples. The water absorption of the reference sample was 7.1 ± 0.1%, while it was 7.2 ± 0.1% for the sample coated by immersion and it was 7.4 ± 0.1% for the sample coated by painting.

#### 2.2.3. Compressive Strength

The compressive strengths of the samples with surface-applied compounds are comparable to the samples without compounds. All tested samples, regardless of the application of the compound and its method, not only met the requirements for 42.5 R grade cement, but also achieved a strength of over 75MPa. The uncoated sample achieved 84.2 ± 1.8 MPa; the sample coated by dipping achieved 76.2 ± 4.8 MPa; and the sample coated by painting achieved 80.6 ± 1.5 MPa. It can be assumed that the compressive strength of the cementitious matrix is not affected by the application of L43 on the surface of the cement samples.

#### 2.2.4. Test of Resistance to Cyclic Freezing and Thawing

**Figure 2:**
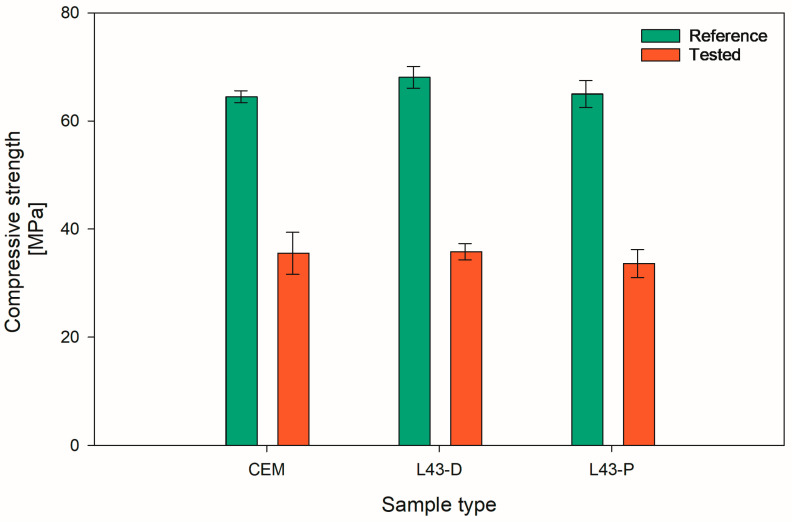
Mean compressive strength after freeze–thaw resistance test of the samples with surface-applied L43.

Frost resistance tests on samples with surface-applied L43 showed no significant differences to samples without the applied compound, and the results are shown in Figure 2.

**Figure 3:**
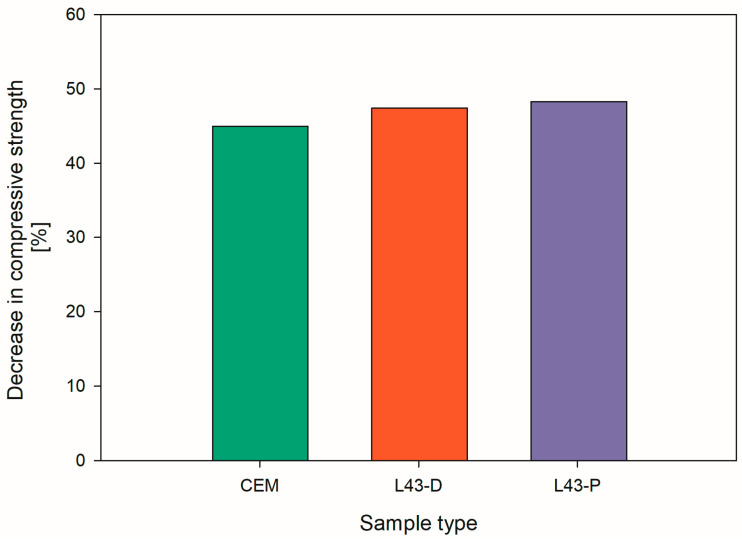
Percentage decrease in compressive strength after freeze–thaw resistance test of the samples with surface-applied L43.

The decrease in strength, shown in Figure 3, after the frost resistance test was about 45%, which was comparable with the specimens not coated with L43 and those coated by both painting and dipping. It can be assumed that the frost resistance of the cementitious matrix is not affected by applying L43 on the surface of the cement samples.

#### 2.2.5. Biological Testing

**Figure 4:**
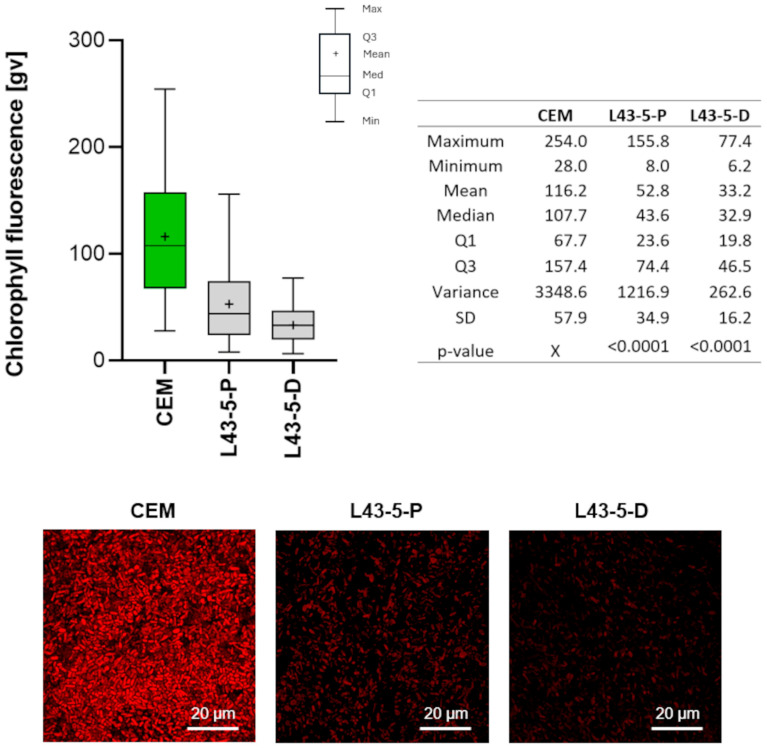
Changes in ^chl^FI in microalgal cells growing for 21 days on cement mortar between control (CEM) and samples covered by the tested compound in 5% concentration by painting (P) and dipping (D).

The L43 compound in 5% concentration coating cement mortar reduced microalgal cells’ photosynthetic activity, and it was clearly visible as the reduction of the fluorescence intensity in the CLSM view (Figure 4). In both cases—the painting and dipping method of compound application—the ^chl^FI significantly decreased in relation to the control (*p* < 0.0001) and to each other (*p* = 0.0001). However, cells grown on mortar covered by dipping in the L43 had a higher reduction rate in the ^chl^FI. Maximum ^chl^FI decreased by 69.5%, while the mean value decreased by 71.4% (Figure 4).

#### 2.2.6. Surface Properties

**Figure 5:**
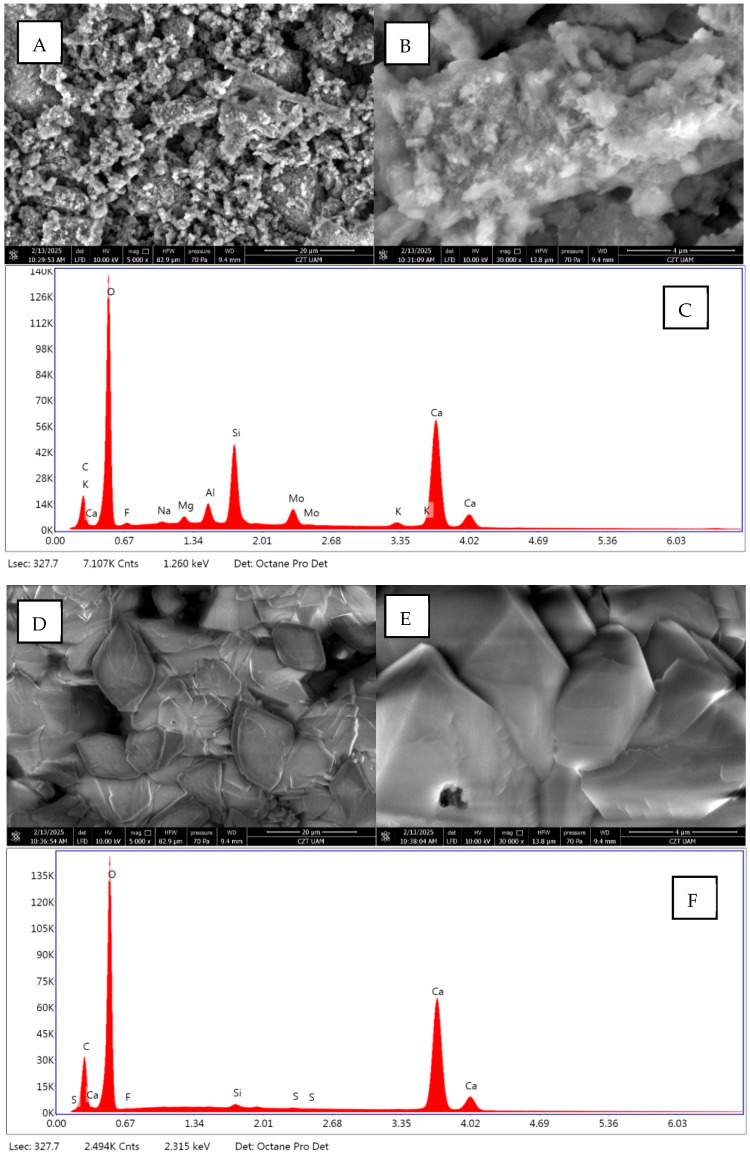
SEM characterization and results of EDS procedure of untreated mortar surfaces (**A**–**C**) and mortar surfaces treated with L43 (**D**–**F**).

The changes in surface morphology and material cohesion after the treatments were observed by SEM. As shown in Figure 5, the untreated cement mortar composite (Figure 5A–C) has a heterogeneous surface where the mineral grains are clearly visible. After treatment with the L43 product (Figure 5D–F), the coating is more compacted and has a defined structure in which the product fills the spaces between the mineral grains, resulting in a more cohesive aspect. It should be noted that no cracking of the coating was observed. X-EDS analysis confirmed that the coating was covering the surface. It must be taken into account that the SEM image was taken on the surface, where the material is likely to be more compact due to the presence of a greater quantity of consolidant than in depth.

**Figure 6:**
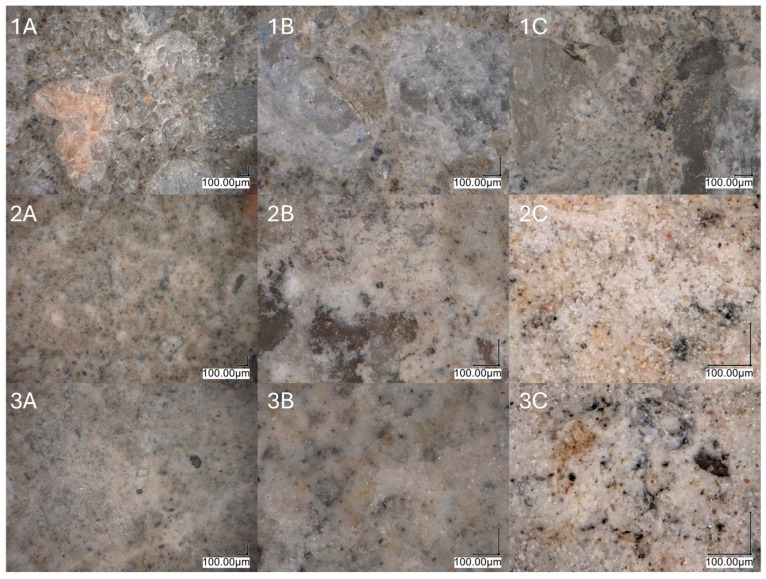
Microscopic images of the reference (**1A**–**1C**) and modified samples: painted, L43_P (**2A**–**2C**); dipped, L43_D (**3A**–**3C**).

Figure 6 presents microscopic images of the composite surfaces. The reference sample (1A–1C) exhibits a smooth surface with visible mineral phases and components. Samples modified by either painting or dipping (2A–3C) contain a modifier layer on the surface. The painting method results in a thicker layer, which contributes to a less uniform morphology and promotes the formation of fine-crystalline deposits, as shown in image 2C. The dipping method, however, facilitates the formation of a more homogeneous layer due to the removal of excess modifier during the process. This leads to a significant reduction in the number of crystalline aggregates on the surface (3A–3C). The observed morphology suggests a more controlled deposition process and better adhesion of the modifying layer.

## 3. Discussion

In terrestrial environments, atmospheric conditions such as wind and rain are essential for the distribution of microalgae from one substrate to another [27,28]. Individual cells break away from well-developed biofilms and spread to new substrates by wind. Once they reach new surfaces, the extracellular polymeric substance they produce helps them to adhere to even very smooth surfaces. At the right humidity, they begin to develop multilayered biofilms consisting of a bacterial–algal matrix that cooperates to survive the harsh conditions of the terrestrial environment [29]. For the experiment, all inoculum mixtures consisted of selected algal strains with their native bacterial microflora. By using this non-axenic culture, there was greater confidence that the algae would adapt to the specific substrates and form biofilms. Moreover, if there is a positive anti-algal effect, this type of culture more closely reflects the ongoing phenomena in the environment where bacteria and algae cooperate in the formation and survival of photosynthetic biofilms. In addition, the control concrete substrate was overgrown with algal cells and biofilms were formed.

The antifouling effect of highly hydrophobic compounds like alkylsilane and alkylsiloxane is well known. Their effect is to minimize the adhesion force between cells, which gives the surface cell-releasing properties [30]. However, hydrophobic surfaces cannot prevent the colonization of fouling organisms. This is because some microalgae secrete extracellular polymeric substances and show an even better ability to colonize hydrophobic surfaces [31]. Hydrophilic compounds, on the other hand, can prevent the colonization of microorganisms in the initial state due to the hydration layer formed [30]. The proposed functionalized siloxane L43 has both hydrophobicity (fluoroalkyl group) and hydrophilicity (hydroxyl group) in its structure. As a result, it acts more like an amphiphilic coating with hydrophobic and hydrophilic elements. The effectiveness of such compounds in controlling algal fouling was confirmed by Wang et al. [32], where the amphiphilic compound showed high effectiveness in reducing spores of marine green algae from the surface of the material. In the air environment, the key factor for cell survival is the availability of water, at least in the form of water vapor in the air. Microalgae in the air are poikilohydric organisms that cannot regulate their own water content. Limited access to water increases the desiccation state of the cells, leading to metabolic disorders. However, it is not only environmental conditions that increase desiccation. There is evidence that hydrophilic surface coatings can also dehydrate algal cells [33]. Analysis of photosynthetic activity for samples of concrete surface protected by painting (P) and dipping (D) with components containing bifunctional siloxane L43 showed statistically significant changes (*p* ≤ 0.05) for both the coatings applied by painting (P) and dipping (D). Data within the 1st and 3rd quadrants were lower than the control sample. A greater decrease in the maximum value of chlorophyll fluorescence compared to the control sample was observed for the sample surface impregnated by dipping (D). It can be concluded that during the application of the coating by immersion (D), a more uniform layer is formed on the cement mortar surface. This results in a significant reduction in the number of crystalline aggregates on the surface, which is confirmed by analysis using an optical microscope. During the dipping process, the excess modifier is naturally removed. The excess preparation flows off the sample after removal from the modifier solution. The observed morphology suggests that the immersion coating (D) process is more controlled and provides better adhesion of the modifying layer.

## 4. Materials and Methods

### 4.1. Materials

#### 4.1.1. Bifunctional Siloxane L43

All commercially available chemicals were used as received without any further purification. 1,1,3,3-Tetramethyldisiloxane was purchased from Gelest (Arlington, VA, USA). Allyl polyether (BIKANOL9) was purchased from ICSO Chemical Production, Kędzierzyn-Koźle, Poland. 1,1,2,2,3,3,4,4-Octafluoropentyl allyl ether was synthesized by the Williamson reaction of octafluoropentanol and allyl chloride. The hydrosilylation catalyst used was commercially available Karstedt’s catalyst, purchased from Merck Life Science Sp. z o.o., Poznan, Poland.

#### 4.1.2. Mortar Composition

The samples were prepared using CEM I 42.5 R, a hydraulic binder produced by co-grinding Portland clinker (the primary component) with sulfate-based additives to regulate setting time. This cement complies with the specifications of EN 197-1 [34]. Chemical properties of cement are presented in Table 1, while the mechanical and physical properties are described in Table 2. Chemical analysis of the composition of CEM I 42.5 R cement is presented in Table 3, and the analysis was carried out by instrumental methods according to the standard PN-EN 196-2:2013 [35]. The quartz sand used as a fine aggregate in the cement mortars has a maximum grain size of 2 mm and meets the requirements of EN 196-1 [36]. Distilled water, conforming to EN 1008 standards [37], was used in mortar preparation.

#### 4.1.3. Mortar Preparation

All mortars were prepared in accordance with PN-EN 196-1 [36], using a standard mix ratio of 1:3:0.5 (cement:sand:water) and a water-to-cement (w/c) ratio of 0.50. Each batch, sufficient for three specimens, contained (450 ± 2) g of cement, (1350 ± 5) g of sand, and (225 ± 1) g of water.

To ensure consistency, materials and equipment were conditioned to laboratory temperature, and all components were weighed with an accuracy of ±1 g. The mixing process began with water, followed by cement, which was mixed at low speed for 30 s. Sand was then gradually added over 30 s. This was followed by 30 s of high-speed mixing, a 1 min 30 s pause, and a final 60 s of high-speed mixing.

**Figure 7:**
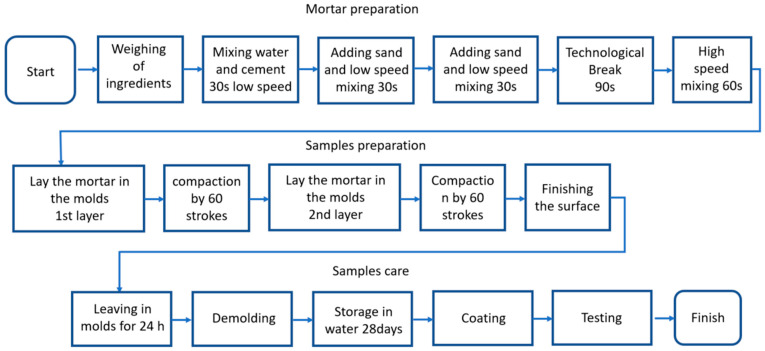
Flow chart of mortar preparation.

The mortar was placed into 40 × 40 × 160 mm molds in two layers, each compacted with 60 shakes. After leveling, the specimens were left to set for 24 h before demolding and curing in water until testing. A flow chart of mortar preparation is shown in Figure 7.

#### 4.1.4. Modification of Specimens with L43 Compound

Cement mortar specimens measuring 40 × 40 × 160 mm were cleaned by removing contaminants from their surface. A 5% solution of the modifier in isopropanol was then applied to the prepared substrate. The surface was impregnated by immersing the specimens in the modifier solution for 30 min or by painting them twice using the wet-on-wet method. The samples were air-dried at room temperature until a constant mass was obtained. They were then subjected to mechanical and biological tests. The test results were compared to an unmodified reference sample.

### 4.2. Methods

#### 4.2.1. Synthesis of Bifunctional Siloxane L43

Siloxanes containing fluorofunctional groups and polyether reactive groups were synthesized by the hydrosilylation reaction of 1,1,3,3-tetramethyldisiloxane and 1,1,2,2,3,3,4,4-octafluoropentyl allyl ether followed by allyl polyether containing nine ethoxy groups with a terminal hydroxyl group. The process was carried out in the presence of Karstedt’s complex as a catalyst. In the first step, 1,1,3,3-tetramethyldisiloxane and 1,1,2,2,3,3,4, 4-octafluoropentyl allyl ether (in a stoichiometric ratio of 1:1) and toluene (in an amount corresponding to half of the total volume of the reaction mixture) were placed in a three-necked round-bottom flask equipped with a thermometer, a reflux condenser and a magnetic stirrer. Karstedt’s catalyst (3 × 10^−5^ mol Pt per mol Si-H) was then added at room temperature. After the introduction of the catalyst, the solution was heated to a temperature of 110 °C. When complete conversion of the fluorinated olefin had occurred (as monitored by FT-IR analysis), an appropriate amount of the second olefin, allyl polyether BIKANOL9 (in a stoichiometric amount to the other substrates of 1:1:1), was added. After all the reagents had been introduced, the solution was kept under the same conditions as before. The course of the reaction was monitored by IR spectroscopy, by observing the disappearance of the band at 904 cm^−1^, which was assigned to the Si-H bond in the substrate. At the end of the process, the post-reaction mixture was cooled and the products were isolated by distilling off the solvent and excess olefin under reduced pressure. The pure product was obtained in a high yield of 98%. NMR analysis of the products confirmed their structure [38].

#### 4.2.2. Water Absorption

A test was used to determine the water absorption of a sample submerged under normal atmospheric pressure, following the PN-B-04500 standard [39]. It was performed on three specimens measuring 40 × 40 × 160 mm. After full saturation, the samples were dried to a constant weight, ensuring that the weight difference between consecutive 24 h intervals did not exceed 0.2%.

#### 4.2.3. Density

The density of the samples was measured in accordance with PN-EN 1015-10 [40]. The test involved weighing and measuring three 40 × 40 × 160 mm specimens to determine their volume and density.

#### 4.2.4. Compressive Strength

The compressive strength test was conducted in accordance with PN-EN 196-1 [36] by using the Servo-Plus Evolution testing machine (Matest S.p.A., Treviolo BG, Italy). First, the 40 × 40 × 160 mm beams were broken during a flexural strength test. The flexural test involves placing the specimen on 2 roller supports with a spacing of 100 mm and then loading the specimen in the middle of its span by gradually increasing the load by 50 ± 10 N/s until breaking. Then, half of the beam was placed in a specialized insert with square compression plates made of hardened steel (40.0 ± 0.1 mm on each side). The plates applied pressure to the central part of the beam. The load was applied uniformly at a rate of 2.4 ± 0.2 kN/s until the specimen failed, Supplementary Material Figures S3–S7.

#### 4.2.5. Frost Resistance Test According to PN-B-06256 [41]

The freeze–thaw test was conducted in accordance with PN-B-06256 [41]. Six pre-weighed, water-saturated, and matured samples were subjected to cyclic air-freezing and water-thawing. Freezing was carried out at −18 ± 2 °C for at least 4 h, followed by thawing at 18 ± 2 °C for 2 to 4 h. Meanwhile, six control specimens were maintained in water or at >90% relative humidity at 18 ± 2 °C. After the designated number of cycles or upon visible damage, all specimens were weighed and tested for compressive strength, including the control samples, Supplementary Material Figures S8 and S9.

### 4.3. Physicochemical Characterization of Bifunctional Siloxane L43

#### 4.3.1. Magnetic Nuclear Resonance Spectra

NMR Spectra (^1^H NMR, ^13^C NMR and ^29^Si NMR) were taken on a Bruker Ascend 400, at room temperature, with CDCl_3_ as a solvent.

#### 4.3.2. Mid-Infrared FT-IR Spectrometer

FT-IR spectra were taken on a Nicolet iS20 Mid-Infrared FT-IR Spectrometer with a Gate diamond ATR attachment. The spectra were collected in the range 500–4000 cm^−1^, with a resolution of 2 cm^−1^, always recording 32 scans of the background and the sample. The progress of the reaction was quantified by observation of the rate of change in the area of the band with a maximum at 904 cm^−1^, assigned to the stretching vibrations of Si-H.

#### 4.3.3. Thermogravimetric Analysis

TGA)was performed on a Q50 apparatus (TA Instruments, New Castle, PA, USA), under nitrogen flow (60 mL/min) from room temperature to 800 °C, at a heating rate of 283.15 K/min (10 °C/min).

#### 4.3.4. Differential Scanning Calorimetry

DSC measurements were performed on a DSC1 instrument (Mettler Toledo, Parramatta, Australia). Analyses were carried out under argon atmosphere blown at a rate 20 mL/min. All samples were carefully weighted and placed in aluminum crucibles. Results were analyzed using STAR^®^ Software (https://www.mt.com/pl/pl/home/library/product-brochures/lab-analytical-instruments/STARe_software_Brochure.html, accessed on 19 May 2025) provided by Mettler Toledo.

Temperature regime:

(1) Isotherm at −80 °C for 5 min;
(2) Heating to 200 °C at a rate of 10 °C/min;
(3) Isotherm at 200 °C for 5 min;
(4) Cooling to −80 °C at a rate of −10 °C/min;

#### 4.3.5. Scanning Electron Microscopy (SEM) Analysis

Scanning electron microscopy (SEM) analyses were performed using a Quanta FEG 250 (FEI) electron microscope with a beam energy of 10 keV. All samples were analyzed without prior preparation. The microscope was operated in low-vacuum mode at a pressure of 60 Pa. The energy dispersive spectroscopy (EDS) analyses were performed using the EDS Octane SDD detector (EDAX). The chamber pressure and beam energy were the same as for SEM imaging.

#### 4.3.6. Surface Structures Analysis

Surface structures were analyzed under a Digital Light Microscope Keyence VHX 7000 with 100× to 1000× VH-Z100T lens (Osaka, Japan). All the pictures were recorded with a VHX 7020 camera, (Osaka, Japan)

#### 4.3.7. Biological Testing

The effectiveness of the L43 compound in limiting microbial colonization of cement mortar was tested using four green microalgal strains that pioneer mineral substrates (stone, concrete, cement, gypsum, etc.) and actively grow in terrestrial environments [13]. The microalgal culture consisted of three coccoid green algae strains in equal proportions: Chlorodium saccharophilum PNK010, Pseudostichococcus monallantoides PNK037, and Trebouxia aggregate PNK080, and a filamentous Klebsormidium flaccidum PNK013/2 was inoculated on the control and treated cement mortar in a volume of 0.5 mL and cultivated for 21 days under laboratory conditions as given in Karasiewicz et al. [33].

The effect of L43 on microalgal cells was analyzed by changes in photosynthetic activity, which directly reflects the physiological state and biological activity of photoautotrophic microorganisms [42]. The biofilm was gently removed from the mortar using a soft sterile brush, and the photosynthetic activity of the cells was measured using chlorophyll pigment fluorescence intensity (chlFI). The intensity was visualized using a Leica TCS SP8 confocal microscope (Lecia Microsystems, Wetzlar, Germany) in the Laboratory of Microscopic Imaging and Specialized Biological Techniques, University of Lodz, and chlFI was measured using LAS-AF 3.3.0.10134 software. Fluorescence excitation was induced by a white light laser at 488 nm, while detection was recorded in the PMT channel at a wavelength of 620–670 nm. For each sample, 225 measurements were performed.

The chlFI data obtained were tested for normal distribution using the Shapiro–Wilk test for normality. Since all data deviated from normal distribution and were independent, the Mann–Whitney U test was used for statistical comparison between two samples, while the Kruskal–Wallis one-way analysis of variance by ranks with post hoc Bonferroni’s correction was used for multigroup testing. Statistical analyses were performed using PQStat v. 1.6.2 software, and graphs were generated using GraphPad Prism 8.0.1 software.

## 5. Conclusions

The innovative approach presented in this publication to the problem of protecting cement composites from the harmful effects of algae is the use of the functionalized organosilicon compound L43, which exhibits amphiphilic properties. Such compounds with are the subject of a rapidly developing scientific field that combines chemistry, biology and physics. This publication proposes the use of the functionalized silicon compound L43 as a modifier in surface application. The structure of the siloxane backbone makes the compound permeable to vapors and gases, which is crucial for the protection of building and construction materials. The coating allows for free air circulation while providing hydrophobicity due to the presence of fluoroalkyl groups. The presence of polyether groups with a terminal hydroxyl group allows the active ingredient to form a permanent bond with the substrate. It should be noted that the use of a functionalized organosilicon compound such as L43 in protective coatings against biological corrosion is also innovative, as it eliminates the need to add harmful biocides to the formulation. The biocidal effect is due to the structure of the siloxane compound and appropriately selected functional groups. It is therefore an environmentally friendly solution.

Furthermore, the results of the mechanical tests clearly showed that, regardless of the impregnation method, the substrate retains its original strength and frost resistance parameters compared to unmodified samples. L43-coated cement specimens achieved compressive strengths exceeding 70 MPa, while specimens subjected to cyclic freeze–thaw tests achieved compressive strengths exceeding 33 MPa. Thus, it was proven that the L43 compound effectively counteracts biological corrosion without deteriorating the impregnated substrate’s structure. It should be noted that this publication presents a pilot study because only Portland cement was used. Further studies are planned to test the effectiveness of compound L43 on other types of cement and at different water–cement ratios W/C.

## 6. Patents

Karasiewicz J, Ślosarczyk A, Thomas M, Olszyński R. Component based on bifunctional siloxane and isopropanol, how to produce the component and how to use it for surface impregnation. P.451000, 2025.

## Figures and Tables

**Table 1:** Chemical properties of cement.

Type of Sample	Chemical Properties				
	SO_3_ [%]	Cl^−^ [%]	Roasting Loss [%]	Insoluble Residues [%]	NaO_eq_ [%]
CEM I 42.5 R	2.63	0.040	2.48	0.68	0.61

**Table 2:** Mechanical and physical properties of cement.

Type of Sample	Mechanical Properties		Physical Properties			
	Compressive Strength After 2 Days [MPa]	Compressive Strength After 28 Days [MPa]	Setting Time [min]	Water to Normal Consistency [%]	Volume Constancy [mm]	Specific Surface [cm^2^/g]
CEM I 42.5 R	25.4	59.3	204	27.9	0.8	3713

**Table 3:** Chemical composition of cement.

Type of Sample	Chemical Components [%]								
	Roasting Loss	SiO_2_	Al_2_O_3_	Fe_2_O_3_	CaO	MgO	SO_3_	NaO_2_	K_2_O
CEM I 42.5 R	<1.0	17.6	4.3	3.9	67.2	1.5	3.8	0.2	0.9

## Data Availability

Data will be provided upon request by the corresponding author.

## Supplementary Materials

The following supporting information can be downloaded at https://www.mdpi.com/article/10.3390/ijms26115052/s1.
