# Supplementary figures and images for: Using an Innovative Bifunctional Siloxane to Protect Cement Composite Surfaces from Biological Corrosion
Source: Int J Mol Sci. 2025 May 23;26(11):5052. doi: 10.3390/ijms26115052 (PMC12155056; doi:10.3390/ijms26115052)

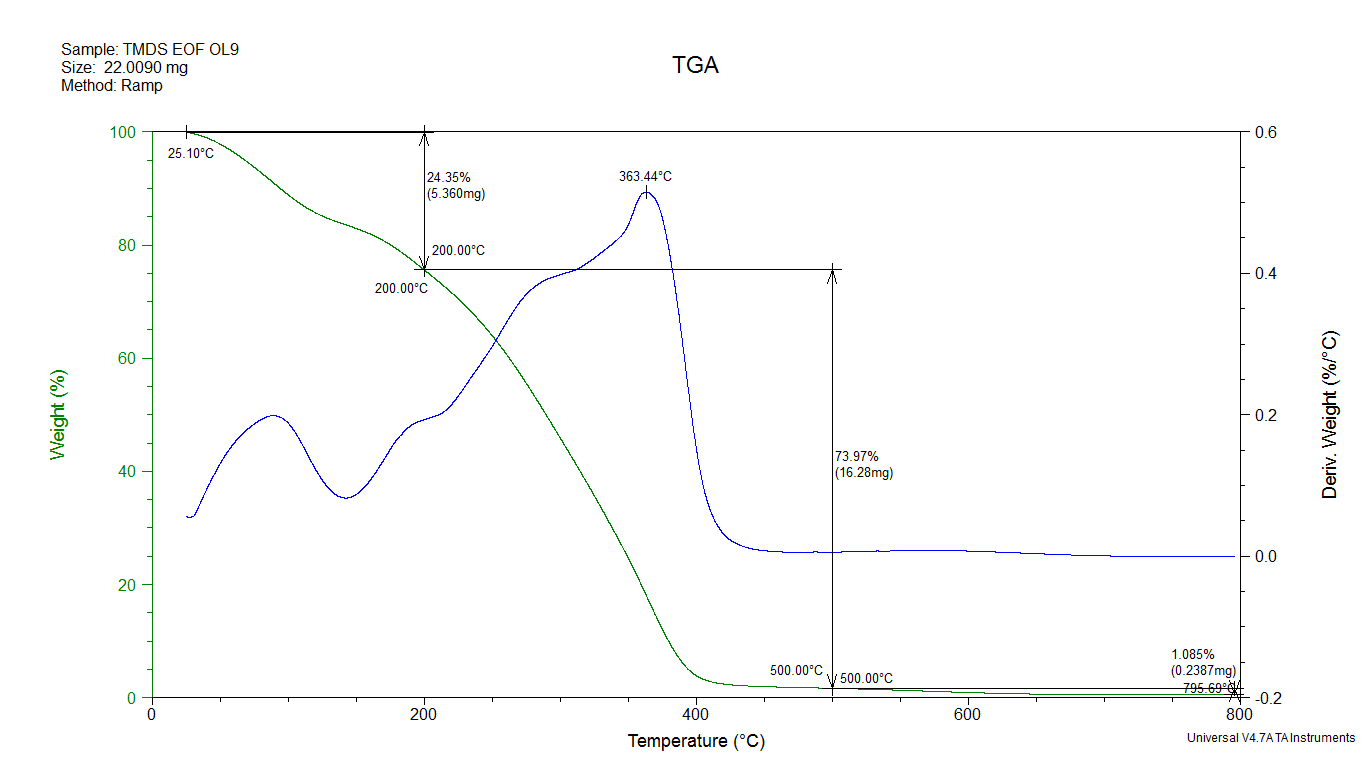

Supplement: Supplementary file 1 [file ijms-26-05052-s001.zip › S1_TG.tif]

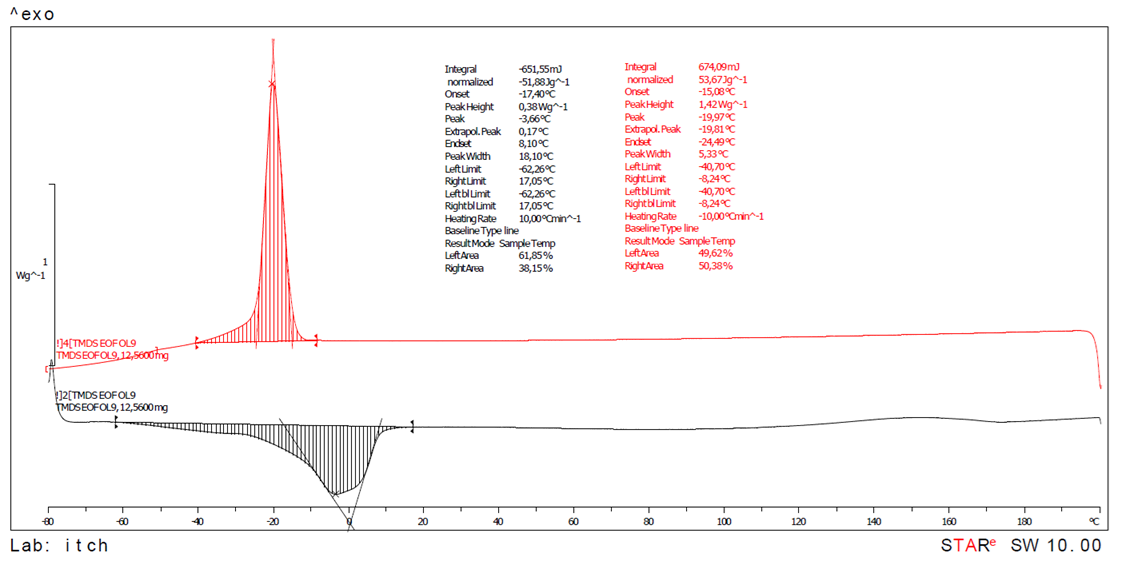

Supplement: Supplementary file 1 [file ijms-26-05052-s001.zip › S2_DSC.tif]

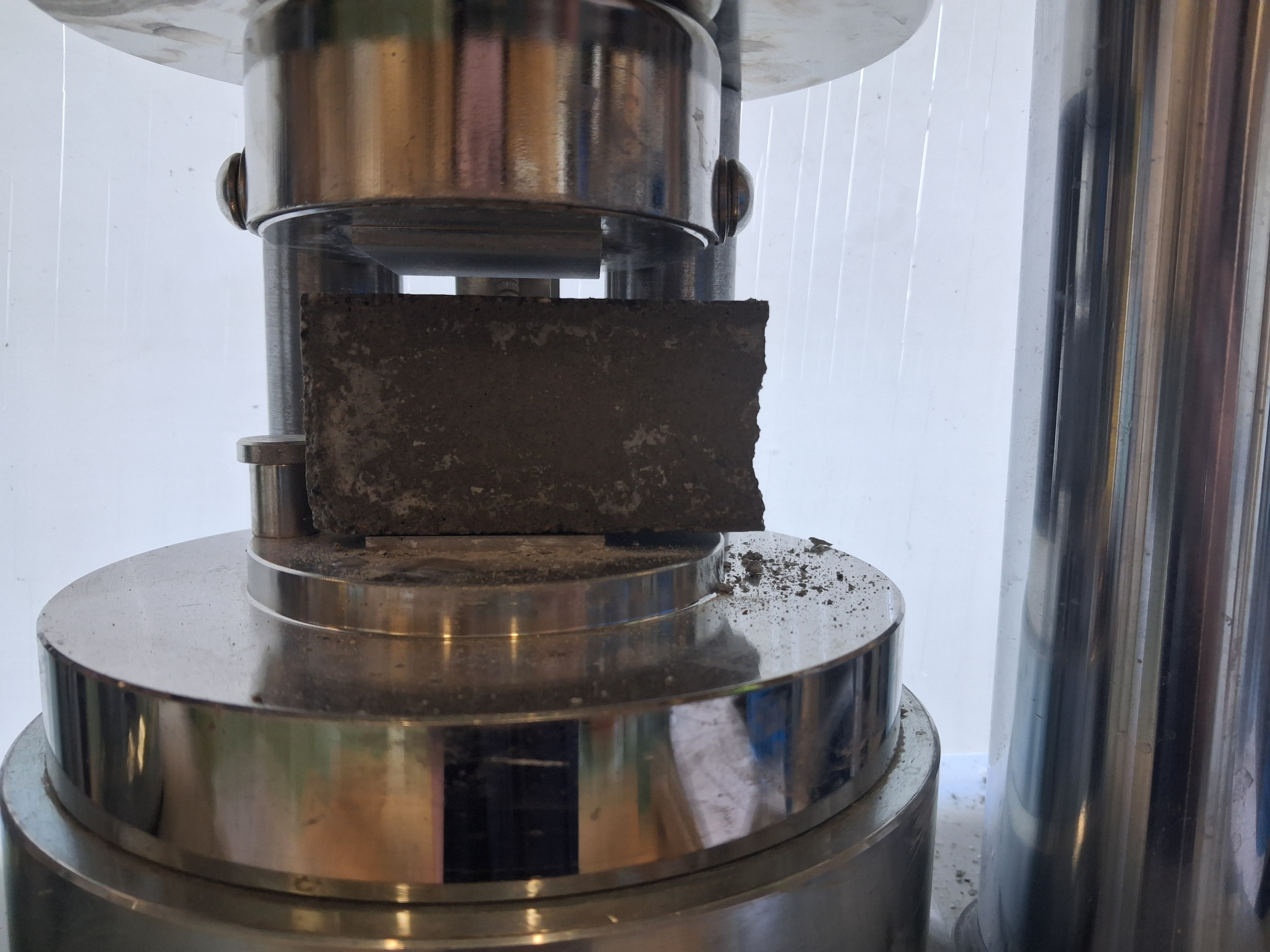

Supplement: Supplementary file 1 [file ijms-26-05052-s001.zip › S3_compressive 1.jpg]

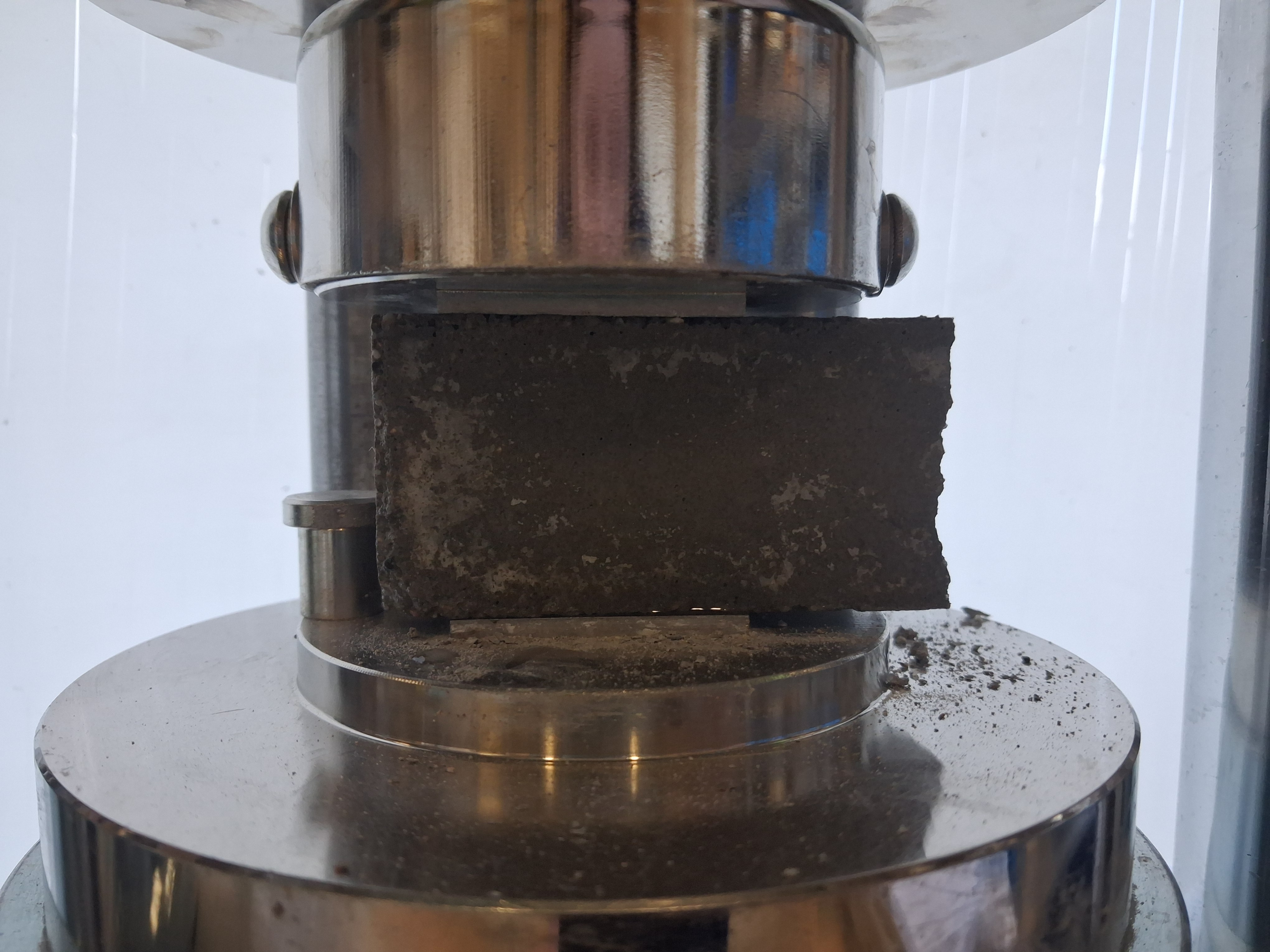

Supplement: Supplementary file 1 [file ijms-26-05052-s001.zip › S4_compressive 2.jpg]

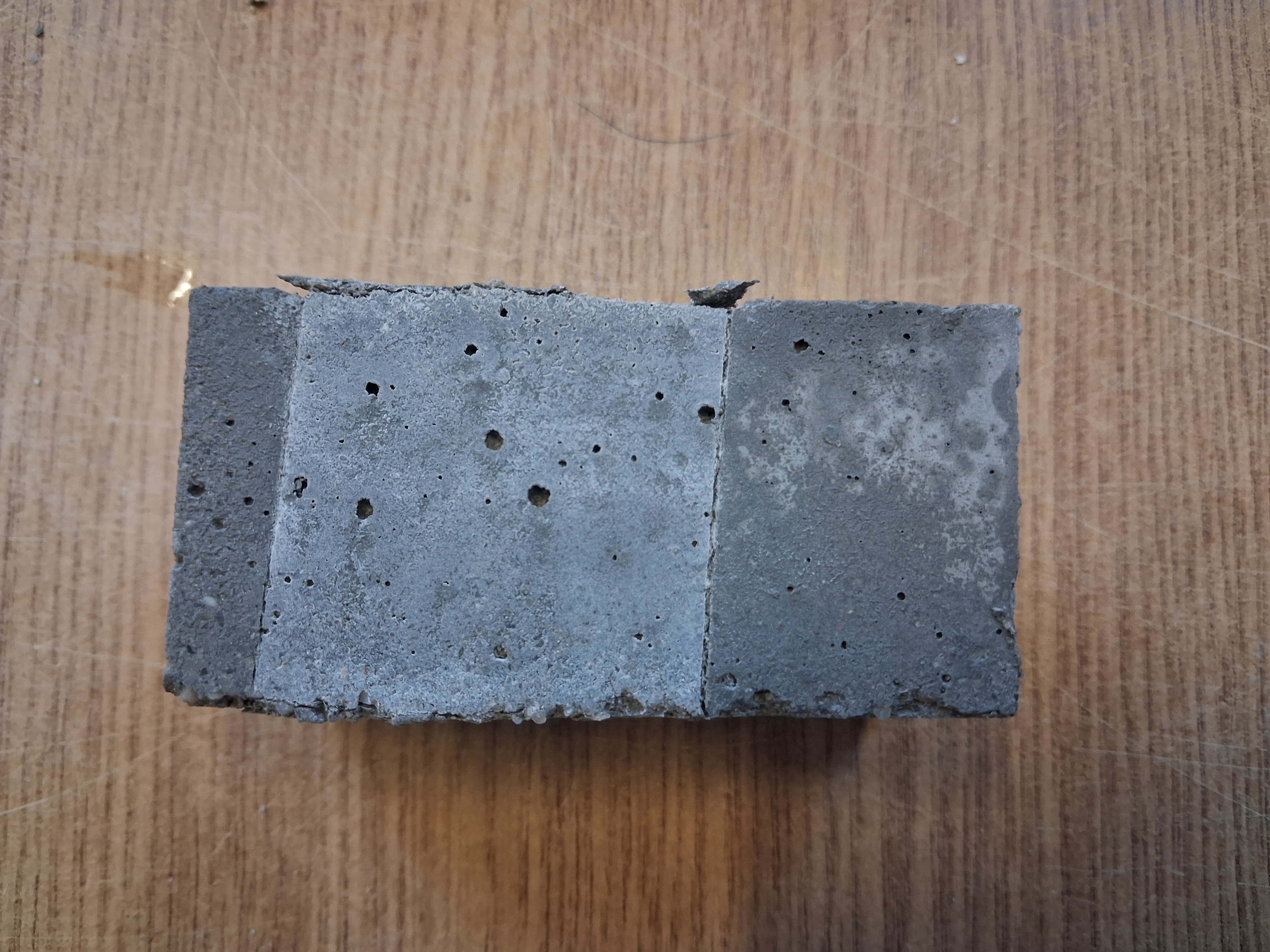

Supplement: Supplementary file 1 [file ijms-26-05052-s001.zip › S5_compressive 3.jpg]

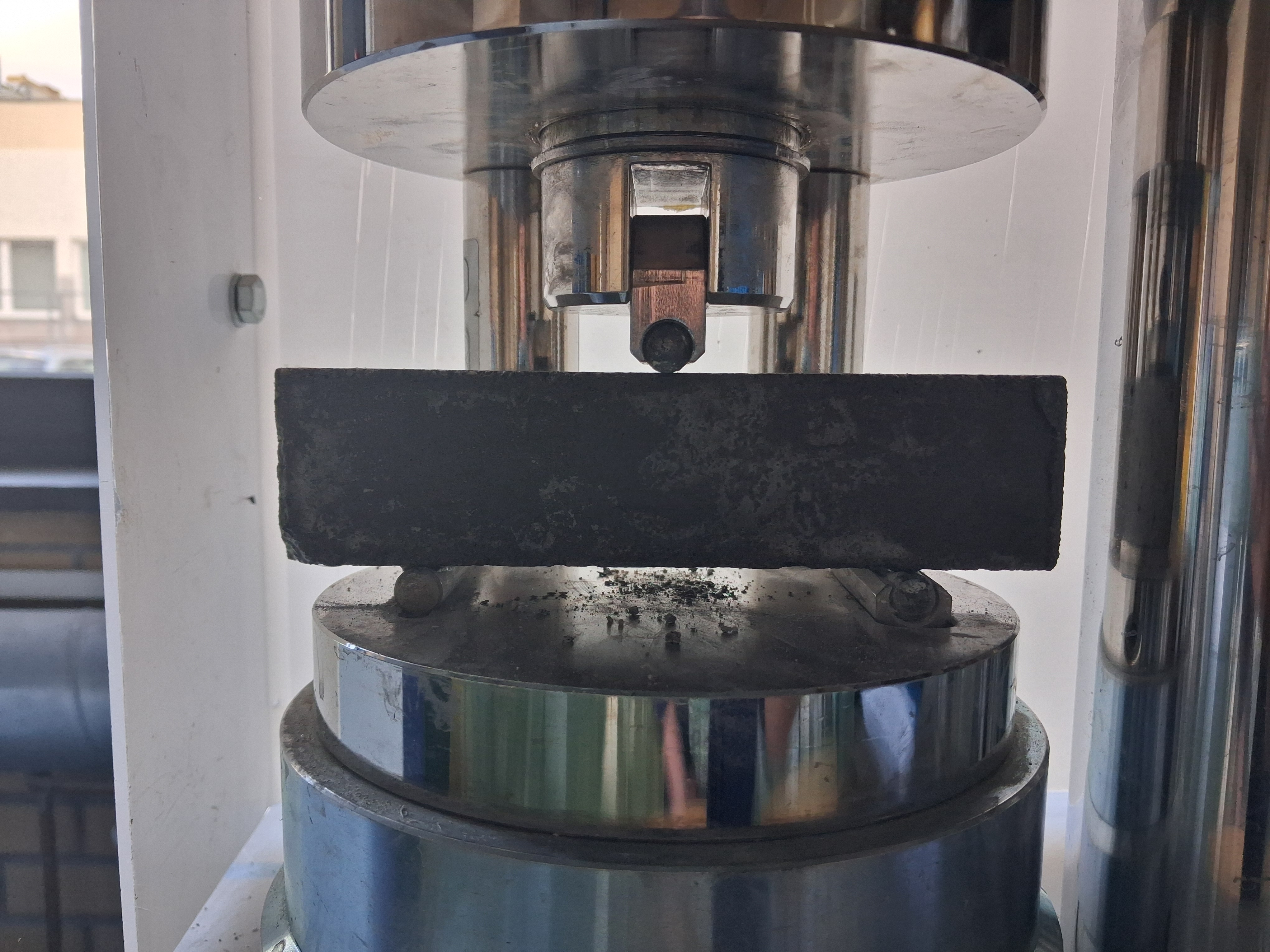

Supplement: Supplementary file 1 [file ijms-26-05052-s001.zip › S6_flexural 1.jpg]

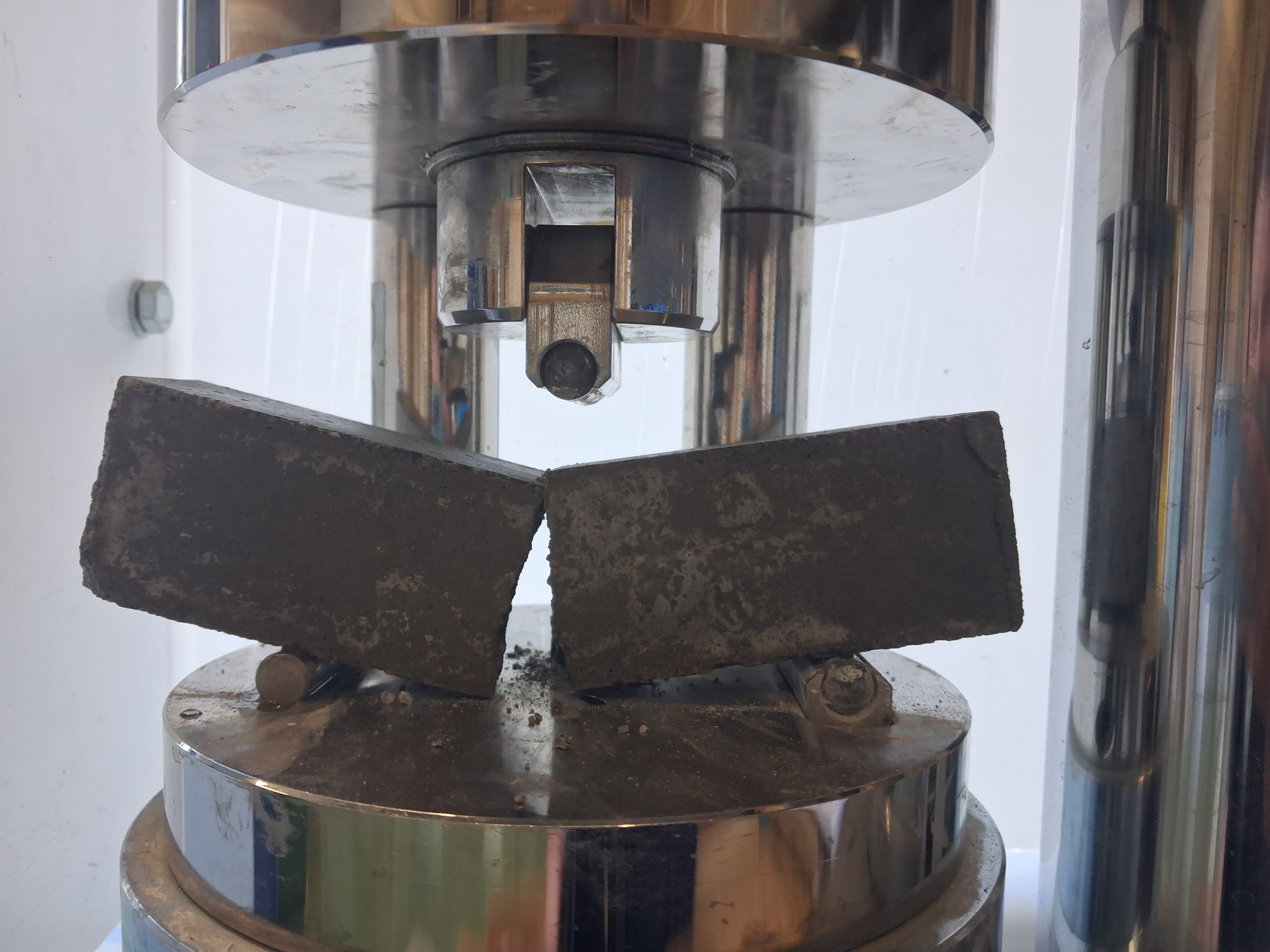

Supplement: Supplementary file 1 [file ijms-26-05052-s001.zip › S7_flexural 2.jpg]

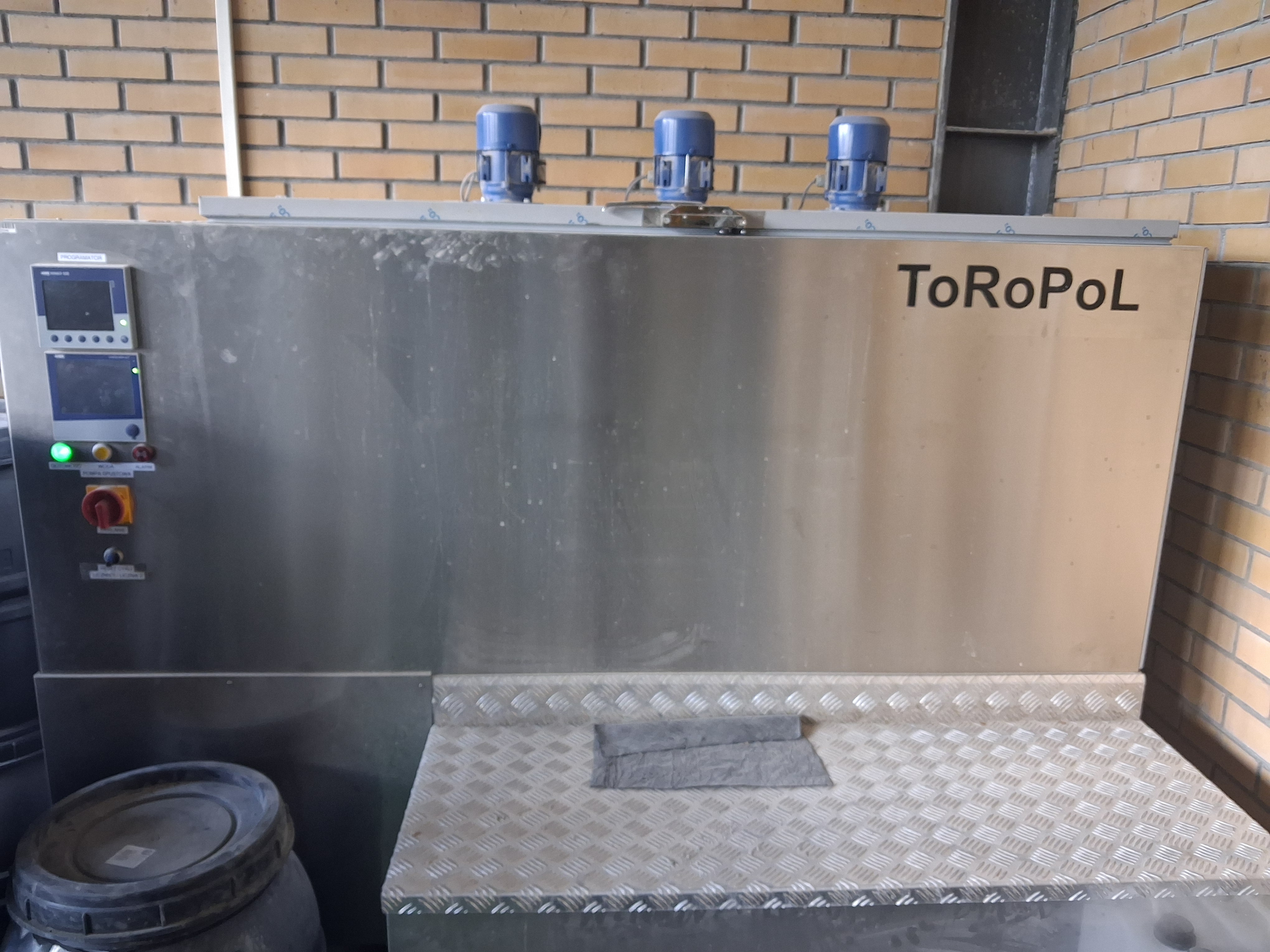

Supplement: Supplementary file 1 [file ijms-26-05052-s001.zip › S8_frost 1.jpg]

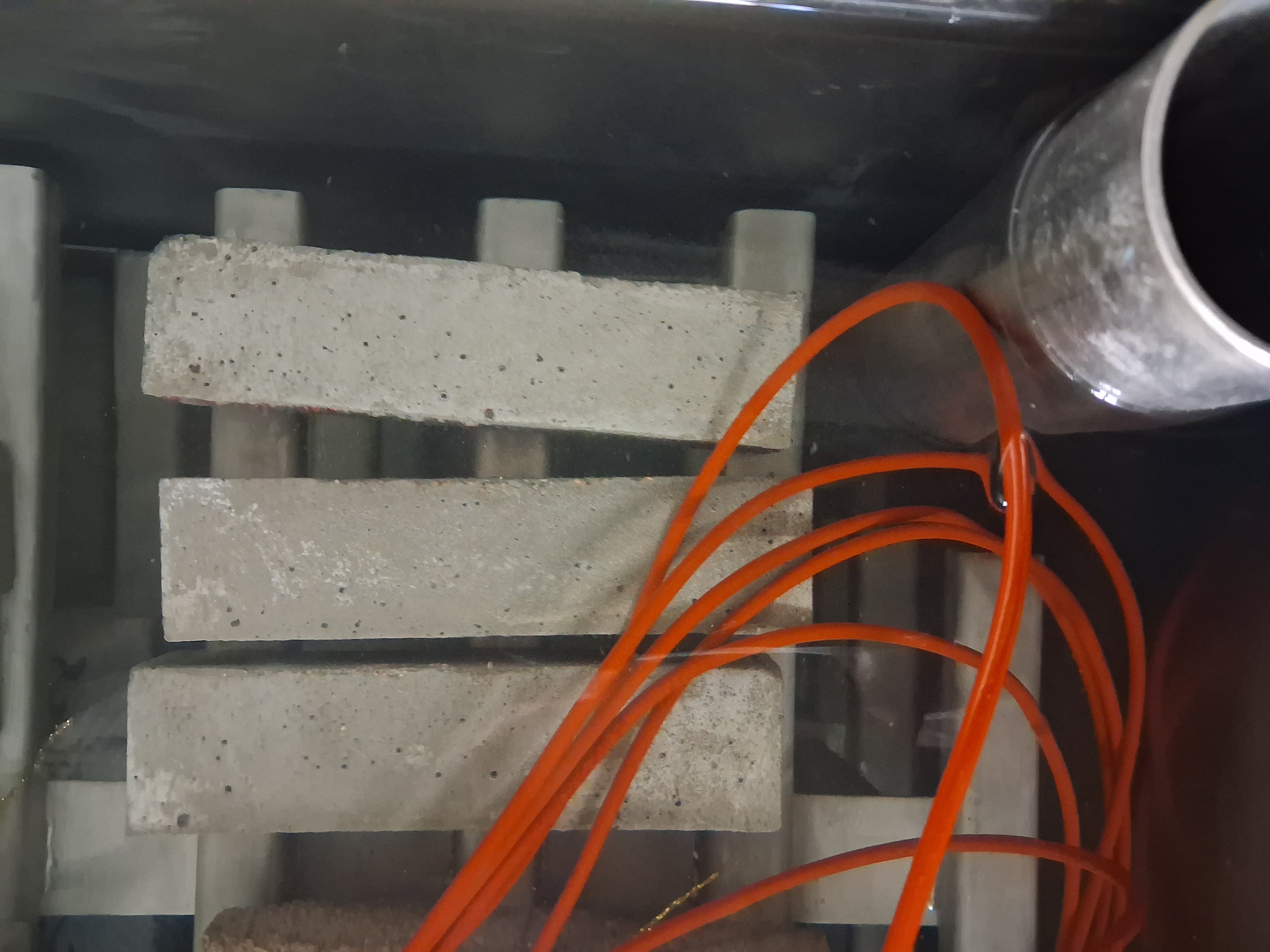

Supplement: Supplementary file 1 [file ijms-26-05052-s001.zip › S9_frost 2.jpg]
